# Development and Pilot Test of a Group Cognitive Behavioral Intervention for Women Recovering From Fistula Repair Surgery in Ethiopia

**DOI:** 10.3389/fpubh.2022.862351

**Published:** 2022-06-06

**Authors:** Tracy R. G. Gladstone, Ana M. Ugueto, Mulu Muleta, Tsega M. Meshesha, Genet G. Ambaafris, Mariya C. Patwa, Cordelia Zhong, Katherine R. Buchholz

**Affiliations:** ^1^Wellesley Centers for Women, Wellesley College, Wellesley, MA, United States; ^2^Department of Psychiatry and Behavioral Sciences, McGovern Medical School, The University of Texas Health Science Center at Houston, Houston, TX, United States; ^3^Ithiel MCH, Addis Ababa, Ethiopia; ^4^MIT Center for Biomedical Innovation, Massachusetts Institute of Technology, Cambridge, MA, United States; ^5^Department of Obstetrics and Gynecology, Saint Paul's Hospital Millennium Medical College, Addis Ababa, Ethiopia; ^6^School of Medicine, Tufts University School of Medicine, Boston, MA, United States

**Keywords:** obstetric fistula, global mental health, anxiety, depression, traumatic stress, cognitive behavioral therapy, psychological treatments, low- and middle-income countries

## Abstract

Obstetric fistula is a serious complication that affects thousands of women in low-income countries. Women who suffer from obstetric fistulae are at risk of developing mental health problems, but to date most interventions have focused on repairing the physical consequences of fistulae through surgery. The goal of the current study is to develop an evidence-based intervention targeting symptoms of depression, anxiety, and trauma in women recovering from fistula repair surgery. First, hospital staff and patients awaiting surgery at a fistula hospital in Ethiopia participated in qualitative interviews to provide information on the mental health needs of women with fistulae, how the hospital tends to these women's psychological needs, and the training needs of staff members. Data from these interviews were used to develop the COFFEE intervention (CBT with Obstetric Fistula for Education and Empowerment). COFFEE is a modular, group intervention that teaches psycho-education, behavioral activation, relaxation, problem solving, cognitive restructuring, and includes a trauma narrative. Patients then participated in an open trial of the COFFEE intervention at the University of Gondar Hospital. Five separate groups were conducted with 24 women who were enrolled post-fistula repair surgery. Women completed pre-treatment self-report questionnaires, participated in group sessions conducted by nurses (with 8 sessions delivered across 10–14 days), and were assessed post-treatment and at 3-month follow-up. Results indicate a significant reduction on depression and anxiety symptoms scores across the three time points [*F*_(2, 40)_ = 68.45, *p* < 0.001 partial η^2^ = 0.774]. Additionally, there was a significant decrease in traumatic stress scores from baseline to post-treatment [*F*_(1.10, 21.98)_ = 100.51, *p* < 0.001 partial η^2^ = 0.834]. Feedback forms completed by nurses and patients suggest the intervention was well-received. Results of this open-trial suggest the COFFEE intervention is feasible, acceptable, and clinically beneficial to treat symptoms of depression, anxiety, and traumatic stress in women post-fistula repair surgery in a hospital setting.

## Introduction

Obstetric fistula, a hole between the vagina and rectum or bladder (i.e., a hole in the birth canal), is a serious obstetric complication generally caused by prolonged obstructed labor, which leads to medical complications for the woman and nearly always results in the death of the neonate (1, 2). While the true rates of obstetric fistulae are difficult to determine due to the lack of reliable population-wide data and inconsistency across prevalence studies (3), early estimates that over 2 million women suffer from this condition worldwide (4) are consistent with the WHO's current estimates for sub-Saharan Africa and South Asia (5), which some contend may even be an underestimate (6). Women who are typically affected by fistulae have underdeveloped pelvic structures or generally possess a small physique (7). Additional factors associated with the development of obstetric fistulae include low socioeconomic status, the cultural practice of early marriage (before age 18), inadequate access to and education about family planning, lack of comprehensive obstetric care, and long distances from home to health care facilities (1, 6, 8).

According to Alio et al. (9), obstetric fistula is associated with a “loss of basic rights as a woman and human being” (p. 376). The consequences of obstetric fistulae have been described as “devastating” and “dehumanizing” (10). As a result of an obstetric fistula, a woman leaks wastes (urine and/or feces), which often results in an odor, burns to the skin along her legs and feet, and progressive complications such as malnourishment, amenorrhea, nerve damage, and foot drop (1, 11). While some women report support from family members, including their husbands (12, 13), many women with obstetric fistulae face traumatizing social challenges. Women with fistulae have described experiencing isolation, feeling shame, losing their jobs, having their food rations decreased, feeling unsupported in hospitals, being unable to use public transportation or participate in community or religious events, and loss of familial and/or martial support (1, 14, 15).

Due to the physical and social challenges that are compounded by limited access to supportive medical care and early surgical repair in low-resource settings, obstetric fistula has significant psychological consequences as well. Women and girls who develop obstetric fistulae are at risk for mental health disorders such as depression, anxiety, and traumatic stress (16–19). Wilson (16) found, relative to gynecology outpatients in Tanzania, obstetric fistula patients reported more psychological distress (i.e., symptoms of depression and PTSD) when entering the clinic, suggesting mental health concerns are particularly salient for these women. In fact, in a study of mental health functioning among women with obstetric fistulae in Ethiopia, all 51 participants screened positive for depression (20). Another review of the prevalence of depression among women with obstetric fistulae in low-income African countries found a pooled prevalence of 56.2%, with women in Ethiopia reporting the highest prevalence of depression at 74.4% (21). Although surgical repair of fistulae is associated with improvements in women's quality of life and mental health (22, 23), many women still face difficulty re-engaging in family and community life, with a range of medical, psychosexual, economic, and psychosocial issues causing further mental health distress post-surgery (24). Muleta et al. (25) found, even after being treated surgically to repair the fistula, many women still had difficulty engaging in family and community life, leading the authors to suggest a need for new strategies to address mental health after fistula repair surgery. Likewise, Mselle et al. (26) found women in Tanzania who were returning home after successful fistula repair surgery remained fearful of isolation and rejection by their communities.

To date, most efforts to address obstetric fistula worldwide have focused on the physical consequences of this problem, yet, according to Muleta et al. (25), “Simply repairing the injuries is not the end of the challenge” (p. 45). Obstetric fistula is a significant problem in low-income countries that is virtually non-existent in high-income countries (1, 27). Unfortunately, few intervention efforts have focused on the psychological impacts of this condition, despite many research groups advocating for integration of mental health care into treatment for women suffering from this condition (17, 18, 22, 24, 28). Overall, women and girls are not taught skills they can use to function effectively and contribute to their communities, to cope with trauma and loss, and to manage every day and more significant life events that they are likely to encounter once they leave the secure environment of the fistula hospital and return home.

The World Health Organization's Mental Health Gap Action Program (mhGAP) recommends the use of evidence-based psychological treatments as the first line treatment for mental health problems (29). Numerous studies have demonstrated the effectiveness of interventions designed to prevent and treat common mental health problems in low- and middle-income countries [LMIC, (30–32)]. A few studies testing the integration of mental health services into fistula care have been implemented and have produced positive results. For example, in one study (33), nurses and public health workers were trained to deliver two individual educational counseling sessions to fistula patients focused on fistula symptoms, surgical care, and discharge instructions; these sessions were associated with short-term increased knowledge about the causes of fistulae and improved self-esteem. Likewise, after participating in group therapy prior to surgery, Ojengbede and colleagues (28) observed a significant reduction in severe depressive symptoms and suicidal ideation, as well as an increase in self-esteem, among women presenting for fistula repair surgery. In addition, Watt et al. (27) reported significant improvement in mental health outcomes over time after offering a six-session individual intervention, delivered by trained community nurses, to women following fistula repair surgery. Yet, despite studies displaying overall quality of life improvement in patients who received psychosocial counseling as part of fistula care, the implementation of these interventions is limited by training, staffing and funding needs (34).

To address these limitations and to improve access to evidence-based psychological interventions, global mental health researchers recommend: (1) using lay counselors (e.g., peers, community workers, nurses) instead of mental health professionals to increase the mental health workforce, (2) training lay counselors in evidence-based treatments (e.g., CBT), (3) providing detailed training and ongoing supervision during the treatment phase, and (4) offering psychological treatments in readily accessible settings (e.g., schools, homes, clinics and hospitals) in the community (32, 35, 36).

These recommendations can be applied to women living with obstetric fistula who are seeking medical care in order to increase access to psychological interventions. Women with fistulae typically travel from rural communities to hospitals in larger cities for repair surgery (14). After receiving surgery, they spend weeks in the hospital recovering (27), which provides a valuable opportunity for psychological treatment. Adding a psychological intervention post-surgery provides more comprehensive care and support for the patients, and can also help the women learn needed coping skills for managing their physical and mental health and for reintegration into their families and communities.

We aimed to develop a culturally appropriate and sensitive CBT group intervention targeting symptoms of depression, anxiety, and trauma in women recovering from fistula repair surgery, which can be delivered by nurses in the hospital during post-operative care. First, we conducted a qualitative study with medical staff and with women who were patients on a fistula unit (both pre- and post-surgery) in a hospital in Gondar, Ethiopia to determine the psychological consequences of fistula, the need for psychological treatments, and the interest by medical staff in delivering a psychological intervention developed specifically for fistula patients post-surgery. We then used information from the qualitative study to develop a CBT intervention and test it in a pilot study.

## Qualitative Study

The goal of this qualitative study was to understand how the mental health of women with obstetric fistulae is addressed in the University of Gondar Hospital's Fistula Center, a 70-bed unit, in order to inform the development of an intervention addressing symptoms of depression, anxiety, and traumatic stress for women recovering from fistula repair surgery. Qualitative surveys were conducted from the perspective of 10 fistula unit staff members (e.g., nurses, residents) to understand their perceptions of mental health, how the hospital tends to women's psychological needs, and the training needs of staff members. In addition, qualitative surveys were conducted with six patients awaiting or just following fistula repair surgery within the hospital to learn about individual experiences with the condition, sources of social support, their understanding and expectations of fistulae and the surgical repair process, and personal desires to learn about and relieve mental distress related to their injury.

## Methods

### Participants

Staff members at the University of Gondar Hospital's Fistula Center were identified by the hospital's chief surgeon (MM) in consultation with the head physician on the unit (GAA) and included any staff members who were present at the time and were interested in participating. A total of 10 staff members participated, including the head physician, the chief resident, 3 additional residents, 3 nurses, 1 intern, and 1 field coordinator that worked locally in the community to support maternal health and fistula prevention. The staff study participants ranged in age from 24 to 36 years, and had worked on the fistula unit from 1 week (the medical intern) to 3 years (the chief resident, head clinical nurse, and the head physician).

In addition, a convenience sample of six female patients who were present on the fistula unit participated in qualitative interviews about their knowledge and experiences regarding fistulae. The women ranged in age from 18 to 40 years (mean age = 28.0 years). While one woman had just developed a fistula after giving birth 2 weeks ago, another woman had been living with a fistula for 10 years (mean time with fistulae = 46.8 months). Two women reported they were married, and the remaining women reported they were single. Three women reported living alone, one woman reported living with her parents and siblings, and two women reported they lived with their husbands. The participants included a student (*n* = 1), reported weaving baskets or cloth (*n* = 3), and/or described themselves as homemakers (*n* = 3) who “cleaned house and fetched water.”

### Procedure

All interview protocols were approved by Institutional Review Boards at Wellesley College, Wellesley, Massachusetts and the University of Gondar, Gondar, Ethiopia. Consent forms were translated into Amharic and read to participants who were unable to read them independently. Interviews with staff members were conducted over the course of 3 days during March 2014 by members of our research team (TRGG, TMM); interviews with patients on the unit were conducted over 2 days during December of 2014 by an Amharic-speaking Ethiopian-American member of our team (TMM), along with a local nurse.

Qualitative interviews with staff study participants lasted for ~20–30 min and were conducted individually in small private rooms on the fistula unit, in English (*n* = 7) or Amharic (*n* = 3). Because the staff members requested not to be recorded, the interviewers took verbatim notes using a structured script with open-ended questions. Staff study participants were given a small lanyard and a pen as a gift for their participation.

Qualitative interviews also were conducted with six fistula patients on the fistula unit. Each interview lasted ~30 min, and each woman received an umbrella as a small gift for her contribution to the study. Interviews were not recorded, as requested by the hospital staff; the interviewer took verbatim notes using a structured script containing open-ended questions.

### Data Analysis

A conventional content analysis approach was used to code interview data for overarching themes (37), separately for data from the medical professionals and the fistula patients. Codes were not mutually exclusive, and each response could contain multiple codes. For both data sets, in the first round of coding, various themes were identified through three authors' close reading of the interviews. Once themes and subthemes were identified, codebooks were developed. Two authors (TRGG, TMM) used these codebooks to individually code the transcribed interviews. The two authors agreed upon the major themes to create a master list, which was then used as the reference for inter-rater reliability, using Miles and Huberman's (38) formula where reliability equals the number of agreements divided by a sum total of agreements and disagreements. Intercoder reliability was based on ratings by a third author (MCP) and was calculated on three of the transcripts from the staff participants, where intercoder reliability was 0.94, and on three of the transcripts from the women with fistulae, where intercoder reliability was 0.95, suggesting an adequate level of agreement.

## Qualitative Themes

### Medical Staff Interviews

Four overarching themes emerged through content analysis of the interviews with staff study participants. These themes include: (1) Stigma of living with fistula, (2) Hospital experiences, (3) Need for mental health intervention, and (4) The role of nurses.

#### Stigma of Living With Fistula

The staff study participants shared their perspective on the experiences their patients with obstetric fistulae have due to their condition. Half of the interviewed staff (5/10) noted the social isolation that the women often face. A clinical nurse shared: “The reality of these women is that they are hidden from their community…they don't tell or talk to anyone else.” A male doctor stated: “Women with fistulae are neglected by the community, their family, because of the smell, their urine.” The field coordinator stated: “Many of these women are divorced, cast out, isolated.”

Staff study participants addressed the devastating realities and stresses, such as isolation and sadness, that fistula patients experience before they receive medical attention, suggesting that an intervention to prevent depression in this population will need to support social skills development and address the trauma associated with this condition.

#### Hospital Experiences

Most staff study participants (8/10) reported three particular forms of support that women find during their time in the hospital: support from medical personnel (including doctors and nurses) who are open to listening, support from the community that is developed between patients and staff, and educational support that is gained through hospital educational sessions. Staff recognized the importance of community in the hospital for women with fistulae who often have never met others with their same medical condition.

The clinic's head nurse shared in detail: “At the hospital, the nurses take care of each woman, we make sure that we understand each and every case. We want to understand and connect with each woman personally—to make her feel at ease… We are the first defense. We ask each woman: ‘How are you? What can I do for you?’… We establish and create a community.”

Two medical residents also shared their insight that: “If they [women with fistula on the unit] realize you're a professional, then they feel free to talk… They do talk, there's a TV room here and they talk with each other in the TV room.”

#### Need for Mental Health Intervention

Nearly all study staff participants (9/10) reported that patients on the unit have significant mental health needs, and nearly half (4/10) believed the patients would be receptive to mental health intervention in the hospital setting. One male doctor reported: “There is a great need, and people would be willing to participate without a doubt.” A clinical nurse concurred, stating: “Of course, they will accept any help… but the issue is the women don't seek help at first because they either don't think anything can be done, or that nothing is medically wrong with them to begin with.” The head nurse reported: “So when we are talking about mental health needs for women with fistulae the need is high and important, necessary, and I feel that the women will willingly accept help… We are aware of the women that are very depressed because we are constantly in contact with the women.” Finally, the medical intern shared: “We need a mental expert here. These women need emotional support even more than the physical.” Staff noted that while they can address the women's physical concerns in the fistula center, they need further training to better support patients' mental health, suggesting the need for a structured intervention approach that can be delivered by individuals without mental health training.

#### The Role of Nurses

Half (5/10) of study staff participants shared information about the role of nurses on the unit as care providers for the women throughout their stay at the hospital. They also commented on the nurses' ability and desire to lead interventions and gain training to enhance emotional support for the patients.

The head nurse shared: “As nurses, we are open to being trained to do anything to make their lives better… Any type of training for nurses would be beneficial for the well-being of the fistula patients.” Another nurse stated: “We [the nurses] want to treat them [the women] but can't do it well because we don't know how to.” The medical intern reported: “The nurses are the primary ones that are trying to help with the emotional needs of the patients here.”

Study staff participants identified fistula nurses as important resources for women on the unit, and nurses expressed a desire to learn strategies to meet women's mental health needs while they are hospitalized.

### Patient Interviews

Four primary themes emerged from the interviews with the six women, including: (1) Coping, (2) Hospital experiences, (3) Expectations related to the surgery, and (4) Interest in mental health treatment. Additionally, several subthemes were noted.

#### Coping

The women shared information about the strategies they used to cope with the psychological effects of their condition. Women noted the benefits of social support, activity scheduling, and distraction techniques. Subthemes extracted from this topic included efforts from family and friends to help them cope with obstetric fistulae, and personal efforts to cope with their condition.

Several women noted that family members and friends offered suggestions about coping with fistulae. Many women (4/6) shared instrumental responses they received from loved ones, such as a 20- year-old homemaker, who noted: “My husband knows of my situation, my disease…He said that I would get better and he had someone take care of me.” An 18-year-old student said: “My family wanted me to get better, so they asked people where I could get treatment… they encouraged me to stay in school.” Half of the women (3/6) also reported unsupportive responses from family, friends, and spouses. For example, another 20-year-old homemaker who developed fistula just 2 weeks prior to the interview reported: “My husband didn't say anything. He said nothing... He hasn't asked about me at all, he hasn't come to see me since I have been here.” Finally, most of the women (5/6) shared support and words of encouragement from loved ones that assisted them in coping with their condition, such as a 40- year-old basket-weaver and tela (traditional beer) vendor, who noted: “[Friends] say ayzosh (take it easy), and that everything will be alright. That I'll be healed, that I'll get better.” Another woman who developed fistula during her third pregnancy, stated: “[My family] say that they are happy I got my health back.”

Many women (4/6) also shared personal efforts to cope with their condition, such as the 30-year-old woman who sells tela and weaves cloth, who said: “I talk to a friend and ask her ‘what should I do?’.” The 40-year old basket–weaver who lives alone in the city shared: “When I feel anxious I talk to my friends, I talk to people.”

Analysis of data from women who were patients on the fistula unit indicates that, before coming to the hospital, they used a range of coping strategies to manage the sadness and isolation they experienced in their home communities, and that an intervention to support their psychological health should include additional coping strategies such as problem-solving and behavioral activation.

#### Hospital Experiences

The women spoke about experiences with hospital staff during their stay. Subthemes for this topic included learning about fistula, meeting others with fistula, and communication with doctors and nurses.

All of the women reported learning about obstetric fistula while in the hospital. A 20- year-old homemaker shared, “Now I have learned how to prevent fistula, by not getting married young and coming to the hospital before a woman goes into labor.” The 40-year-old basket–weaver also attributed her new understanding to the nursing staff: “Since I have been here for a week now I have learned a lot from the nurses during the classes.” Most of the women (5/6) reported limited knowledge about obstetric fistula prior to visiting the hospital. One 18-year-old student stated, “I didn't know anything about fistula before I came here [fistula center].”

All of the women revealed that the hospital gave them the opportunity to meet others with the same condition for the first time. A 20-year-old homemaker shared: “I didn't know anyone else with this problem.” A 30-year-old tela vendor and cloth weaver stated: “Before I came here [fistula center] I didn't know women that had fistula. I was the only one in my village.”

All of the women shared their desires to communicate with medical staff, but some expressed more openness than others. The 30-year-old tela vendor and cloth weaver shared, “I talk honestly with the doctors and the nurses. I am not scared of them, or shy to not tell them anything.” The 40-year old basket weaver reported: “I want to talk to the nurses and doctors about my problems, but sometimes I don't say everything that bothers me.”

At the fistula center, patients reported feeling supported by nurses and other women in the hospital, and learning more about their condition and how to prevent fistulae in the future.

#### Expectations for Their Lives Following Surgery

The women reflected on how fistula repair surgery could impact their lives. Two subthemes related to this topic included women's expectations regarding the outcomes of the surgery, and a desire to share new knowledge about fistulae with others after surgery.

All of the women revealed high expectations for the surgical repair. For example, a 20-year-old homemaker said, “Once I have the surgery, I will care for myself, I will take care of my life.” But half of the women (3/6) also acknowledged the possibility that they would not be cured after surgery. For example, a 20-year-old homemaker said, “If I wasn't cured, I'd stay home and sit alone,” and similarly, a 40-year-old basket-weaver said, “If I'm not cured, I'll do what God has planned for me.”

Assuming a successful surgery, most (5/6) of the women shared a range of post-surgical plans upon returning to their villages. For example, one woman who recently developed fistula stated, “I want to divorce him [her husband]. He's not worth it.” In contrast, a 40-year-old basket-weaver shared, “If I can, I want to get married, I would love to get married.” Most of the women also spoke about plans related to work or school following surgery, such as the 18-year old student who shared: “[I want to] become better so I can go back to school and work with my parents during my free time.”

Some (2/6) of the women reported plans to share their new understanding about fistulae with their home communities. The 18-year-old student stated: “I have to tell others to come if they are in distress during childbirth because you can develop fistula when you have children at a young age.”

#### Interest in Intervention

Several women expressed an interest in learning to manage their stress and other negative feelings related to their condition. All of the women shared their interests in a mental health intervention. The fabric-weaver shared: “I'm interested in becoming better, I'm interested in learning.” Likewise, the basket-weaver stated: “I want to learn how to fix my problems, my worries.” Finally, the 20-year-old homemaker noted: “I would like to learn how to prevent depression and sadness.” Patients expressed optimism about their future post-surgery and expressed a desire to learn strategies for managing depressive symptoms and negative thoughts associated with their medical condition.

## Intervention Development and Pilot Study

Based on the results from the qualitative study, we developed a CBT-based intervention to reduce symptoms of anxiety, depression, and trauma and to support healthy functioning in women recovering from fistula repair surgery. We aimed to evaluate: (1) the feasibility of training nurses in a group CBT intervention, the feasibility of nurses administering the intervention in addition to their regular nursing duties, and the feasibility of patients completing the intervention while recovering from surgery, (2) the acceptability of the intervention to patients and to nurses, and (3) the potential clinical benefit of the intervention in reducing symptoms of depression, anxiety, and traumatic stress in patients immediately after completing the intervention and 3 months later.

## Methods

### Protocol Development

**Figure 1:**
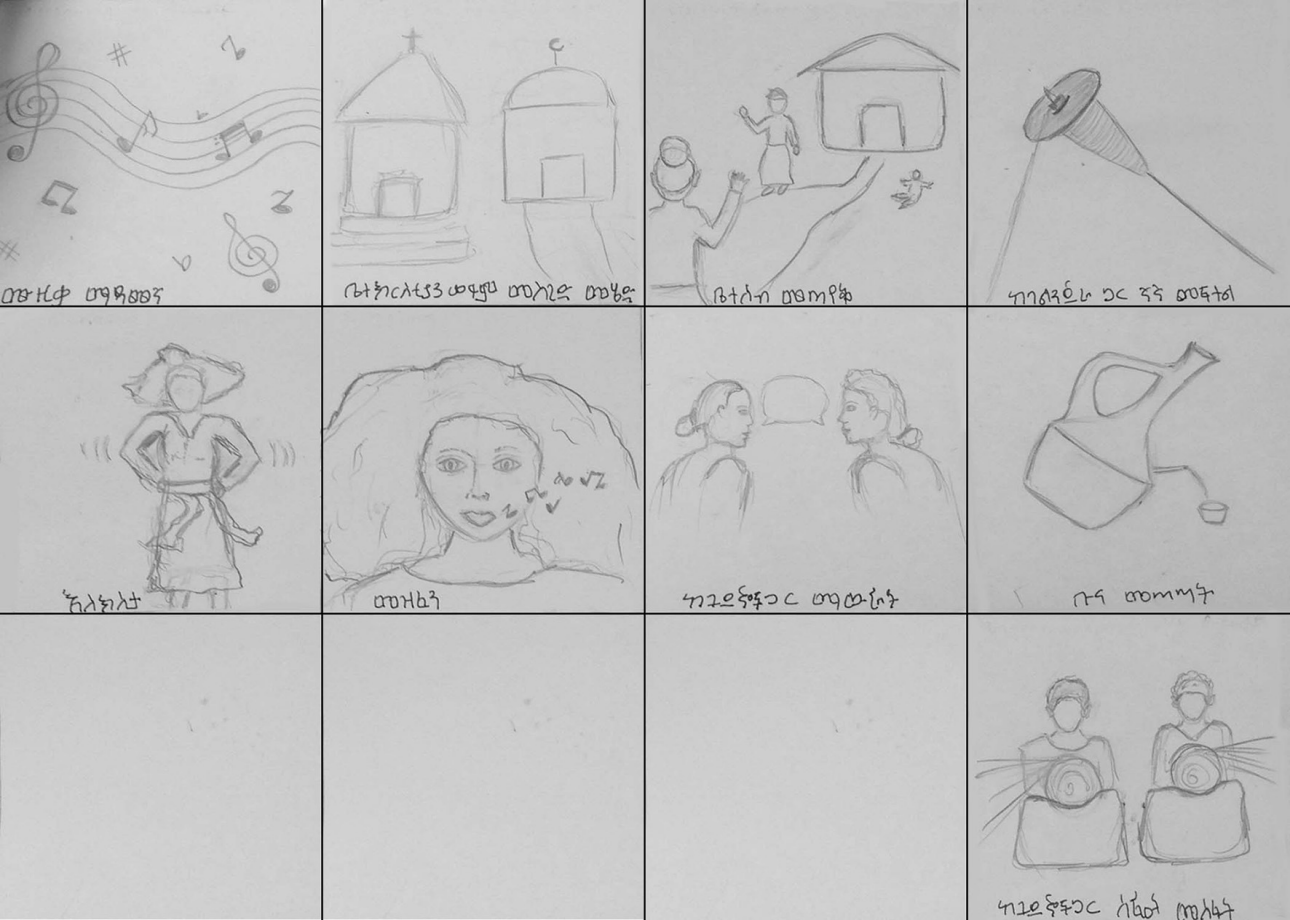
Drawings of fun activities for behavioral activation module.

A group, module-based CBT manual was created specifically for women with obstetric fistulae who were experiencing symptoms of depression, anxiety, and traumatic stress. Cognitive behavioral therapy is an effective, evidence-based treatment, which has been successfully implemented in LMIC by lay counselors (39) and has been used with obstetric fistula patients in Tanzania (27). Common components of CBT for depression, anxiety, and traumatic stress were identified and repackaged for use with women post fistula-repair surgery. The common components, or practice elements, identified included: Psychoeducation, Behavioral Activation, Relaxation, Trauma Narrative, Cognitive Restructuring, and Problem Solving. Three additional sessions were devoted to introducing the treatment, reviewing all of the modules, and an end of treatment celebration (see Table 1 for details on sessions). The manual was designed similarly to other module-based CBT manuals (40, 41) and was adapted to Ethiopian culture through the use of stories, metaphors, and activities (see Table 2). The treatment was named COFFEE, CBT for Obstetric Fistula for Education and Empowerment, because coffee is a staple of Ethiopian culture and “coffee ceremonies” are a source of connection with others in local communities. As many women with obstetric fistulae are illiterate and typically have limited schooling, the majority of didactics were based in discussion and hands on activities; handouts were used sparingly, and emphasis was placed on describing topics in pictures rather than words (see Figure 1).

**Table 1:** Fistula group CBT session descriptions and skills.

**Group session**	**Module**	**Description of module skills**
1	Introduction	• Welcome group members • Explain how obstetric fistulae may affect women emotionally/psychologically • Explain how a group works • Discuss group safety & confidentiality • Explain expectations for group members
2	Psychoeducation	• Explain what thoughts, feelings, and behaviors are • Teach group members how to rate intensity of feelings • Explore connection among thoughts, feelings, and behaviors • Practice changing thoughts
3	Behavioral activation	• Discuss connection between behaviors and feelings • Discuss benefits of engaging in rewarding activities • Make a list of rewarding activities • Engage in fun activity and rate mood before/after
4	Relaxation	• Discuss stress and benefits of relaxation • Teach 3 types of relaxation: Diaphragmatic breathing, imagery, and muscle relaxation • Practice each type of relaxation and rate mood before/after
5	Problem solving	• Discuss how solving problems is a way to improve mood and change behaviors • Practice sequential problem solving and rate mood before/after
(Optional individual session)	Trauma narrative	• Discuss importance of sharing story of trauma • Patient shares story of prolonged labor, death of child • Group leader validates and queries for thoughts, feelings, behaviors and more details • Story is read multiple times and rate mood before/after • Write final chapter about future
6	Cognitive restructuring	• Review connection among thoughts, feelings, behavior • Discuss how changing unhelpful thoughts into helpful thoughts can improve mood • Teach different techniques to change thoughts • Practice changing thoughts and rate mood before/after
7	Review & planning	• Review previously learned skills • Discuss using beads/bracelet as a way to remember skills • Discuss practicing skills at home to manage mood • Plan celebration for next session
8	Celebration	• Group leader praises patients for attending groups and learning new skills • Distribution of certificates • Celebration activity

**Table 2:** Cultural adaptations of behavioral activation, relaxation, problem solving, end of session activities, and celebration.

**Behavioral activation**	**Relaxation (Imagery)**	**Problem solving**	**End of session activities**	**End of treatment celebration**
• Dancing • Singing • Listening to music • Listening to spiritual music • Praying • Going to church • Traditional coffee ceremony • Talking with friends • Playing games • Cleaning house • Helping others • Eating with friends • Weaving with friends or alone • Spinning cotton with friends or alone	• Birds flying • Sunset • River • Moon • Fountain • Breastfeeding a baby • Children playing	• Problems in the hospital: sleeping • Problems at home: family planning, access to medical care, employment/ finances	• Singing a song • Dancing • Telling a story • Telling a joke	• Traditional coffee ceremony • Dancing • Playing/listening to music

COFFEE was designed to be delivered by nurses working on the fistula unit. The group format was chosen to reduce the burden on nurses who were facilitating the intervention in addition to their regular nursing duties. Additionally, the fistula unit is an open ward where patients live side-by-side for numerous weeks, and a group program was believed to capitalize on the friendship and support the women had for each other as well as the collectivist nature of Ethiopian culture. Sessions were conducted in groups with the exception of the Trauma Narrative session. If a woman had elevated symptoms of traumatic stress, she received an individual session because it was thought it would be easier for the woman to share personal details of labor and loss individually and also because it would be difficult for the nurse leading the group to help multiple women create and habituate to a trauma narrative in the same session. Thus, the group leader met individually with the participant and spent as much time as necessary for the woman to share her story in detail and to habituate to the trauma narrative. Previous research in LMIC has found as little as two trauma native sessions can be beneficial in reducing symptoms of trauma (41).

### Group Format

Each group was led by one nurse and followed a standardized format. Group began with a greeting, review of homework, then led to discussion and practice of the new skill, followed by homework assignment, and ended with a fun activity (e.g., singing a song). Homework included discussing how the patients could use the skill during their hospitalization as well as how the skill could be useful when patients returned home to their communities. Groups were designed to be ~60–90 min. Additionally, to help the women remember the different skills after treatment concluded, women were given a colored bead at the end of each session, so by the end of treatment they had a bracelet of different colored beads. The color of the bead also corresponded with the color used in the manual for a particular skill. All groups were held in the community room on the unit.

### Setting

This open-trial was conducted in Gondar, Ethiopia at the University of Gondar Hospital. Gondar is a city in northern Ethiopia with ~325,000 people. Located in the Amhara region, the primary ethnic group is Amhara, and the primary language spoken is Amharic. The majority of people practice Orthodox Ethiopian Christianity. The University of Gondar is the oldest medical school in Ethiopia and houses the Fistula Center, a 70-bed unit with two operating rooms devoted to the surgical repair of obstetric fistula. Surgeons, nurses, and technicians staff the unit. Typically, women from across the northern part of Ethiopia come to the hospital when they are able; once there is a group of about 8–10 women on the unit, about once every 6–8 weeks, a surgeon travels from Addis Abba to conduct the surgeries. The unit opened in January 2010 and by March 2017, over 2000 women had received fistula repair surgeries.

### Group Participants

All women who had received fistula repair surgery and were at least age 18 were eligible and invited to participate in the COFFEE program. All women invited to participate agreed to partake in the COFFEE intervention. Twenty-four women across five cohorts participated in the program between April and September 2015. On average, group members were 27.7 years old (range: 20–50 years), and had lived with obstetric fistula for 50.38 months (range: 1.7 months−20 years).

### Group Leaders

Nurses on the fistula unit were invited to participate in the group treatment by unit leadership. Five nurses delivered the group intervention on the unit. All of the nurses were female and held nursing degrees; one was the head of nursing on the unit, one was a psychiatric nurse, and three were general care nurses. None of the nurses had prior education or experience delivering therapy or psychosocial interventions. Nurses completed the training and provided the group intervention in addition to their regular nursing duties; nurses were compensated for their time assisting with the COFFEE study.

### Study Procedures

After receiving fistula repair surgery, women typically recover in the hospital for 2 weeks, which provides an opportunity for them to participate in the COFFEE intervention. Women were recruited for the intervention after surgery, and then began treatment the following day. The eight sessions of COFFEE were delivered in the 2 weeks after the women received surgery, either occurring every day or every other day based on the availability and the responsibilities of the nurse leading the group. Women provided written consent to treatment before completing the pre-treatment assessment. As many of the group members had difficulty reading, a nurse read aloud the consent form to participants. The study was approved by the Institutional Review Boards at Wellesley College and at The University of Gondar.

### Treatment Fidelity

Nurses completed a 5-day training with the two treatment developers (TRGG and AMU) in March 2015. Each training day was ~8 h and consisted of didactics, modeling of skills by treatment developers, role-plays by group members, group activities, homework assignments, and daily quizzes. Two interpreters were present during the training to interpret for the treatment developers and group leaders; many of the nurses reported speaking or knowing some English. Each nurse was provided with a manual translated into Amharic to use during the training and delivery of the intervention. The manual was written for lay counselors who had no previous psychotherapy experience. The manual provided easy to read, step-by-step instructions on the delivery of each skill, provided sample scripts of how to discuss information, and outlined specific group activities and homework assignments for each session.

During the intervention phase, the nurse who was leading the group intervention spoke with one of the two treatment developers, with the help of an interpreter, after sessions via Skype to review the progress of the group, discuss problems encountered during the group, and to plan for the upcoming group meeting. After each session, the nurse completed a fidelity checklist specific to the group topic assessing if different parts of group (i.e., greeting, homework review, group activity) were completed, and emailed the checklist to the treatment developers before the scheduled call.

### Experimental Design

This study was an open trial (pre-/post-treatment design) developed to assess how COFFEE could help women recovering from fistula repair surgery in a hospital setting. The study assessed the feasibility of nurses implementing COFFEE in a hospital setting with patients' post-surgery, and the acceptability of COFFEE to women who received the treatment and to the nurses who delivered the treatment during routine care. Changes in clinical measures from pre- to post-treatment and post-treatment to 3-month follow-up assessed if the COFFEE intervention was effective in reducing symptoms of depression, anxiety, and trauma, and determined readiness for a full randomized clinical trial.

### Measurement Model

#### Symptom Measures

Women completed the following measures post- surgery and before treatment began, at the end of the treatment, and at 3- month follow-up (Table 3). All measures have previously been used to assess depression, anxiety, and trauma in Ethiopia and have been translated into Amharic. A nurse, who was not leading the intervention group, read the measures to any patient who could not read Amharic at pre- and post-intervention and at 3-month follow-up.

**Table 3:** Assessments and instruments.

	**Post-surgery/Pre-treatment**	**End of each group session**	**End of treatment**	**3-month follow-up**
Self-reporting questionnaire (SRQ-20)	GP		GP	GP
Harvard trauma questionnaire (HTQ)	GP		GP	GP
Group member acceptability			GP	
Group leader fidelity		GL		
Group leader acceptability			GL	

GP, Group Participant^*^; GL, Group Leader. ^*^Assessments read aloud to participants by a nurse who was not their group leader.

##### Self-Reporting Questionnaire

Self-reporting questionnaire [SRQ-20; (42)] was developed by the World Health Organization to assess depression, anxiety, and somatic symptoms in low- and middle-income countries. The SRQ-20 is comprised of 20 items. Respondents answer *yes* or *no* to each item. Scores equal to or higher than 7 suggests depression and anxiety have been present in the past month. The SRQ-20 has been translated into Amharic and validated previously with Ethiopian populations. Internal consistency across studies was good (Cronbach's alpha = 0.84–0.88) (43, 44). In the current study, internal consistency was acceptable (Cronbach's alpha =0.79).

##### Harvard Trauma Questionnaire

Harvard trauma questionnaire [HTQ; (45)] was developed by the Harvard Program in Refugee Trauma. The HTQ is comprised of 40 items that measure exposure to stressful life events over the course of an individual's life. Respondents answer each item on a 5-point Likert scale ranging from *none of the time* (0) to *almost all of the time* (4). The total score is the average score on the questionnaire. Total scores of two or higher indicate traumatic stress, and scores of 2.5 or higher indicate post-traumatic stress disorder. The HTQ has been translated into Amharic and validated with Ethiopian populations (46). In the current study, internal consistency was good (Cronbach's alpha = 0.86).

#### Feasibility and Acceptability Measures

##### Group Member Acceptability

At the end of treatment, women completed a brief 9-item measure about the acceptability of the group. Women were asked to rate how much they enjoyed being in the group, how helpful the group was to learning new coping skills, how engaged they were in group, and how much they understood group content on a scale ranging from 0 (“not at all”) to 10 (“very helpful” or “very much”). Patients were also asked if the beads were helpful in remembering the skills, if they thought other fistula patients would benefit from the program, and if they thought nurses should be trained in the intervention.

##### Group Leader Fidelity and Acceptability

Therapists completed a session-specific fidelity checklist at the end of every session and a treatment acceptability measure at the end of treatment. The module-specific fidelity checklist assesses if specific parts of the module were taught (e.g., review homework, teach skill, assign homework) and also assesses group member engagement and understanding of content. Group leaders also completed a brief 11-item measure about the acceptability of the group to the group leader and the patients. Nurses were asked to rate how much they enjoyed leading the group, how much the patients enjoyed being in the group, how helpful the group was to learning new coping skills, how engaged patients were in group, and how much the group members understood group content on a scale ranging from 0 (“not at all”) to 10 (“very helpful” or “very much”). Group leaders were also asked if the beads were helpful for patients to remember the skills, if they thought other fistula patients would benefit from the program, and if they thought other nurses should be trained in the intervention.

## Results

### Treatment Process and Engagement

All 24 women attended the eight sessions (100% attendance) and completed treatment (100% treatment completion). Based on the results of the HTQ, five patients (20.83%), one patient in each group received a trauma narrative session individually. All eight sessions planned occurred for every group. On average, each treatment group was comprised of 5 group members, each group session lasted 2–3 h, and each treatment was delivered over 10–14 days. All participants completed measures for the pre- and post-treatment assessment points, and twenty-one (88%) participants completed the 3-month follow-up assessment.

### Changes in Symptoms

**Figure 2:**
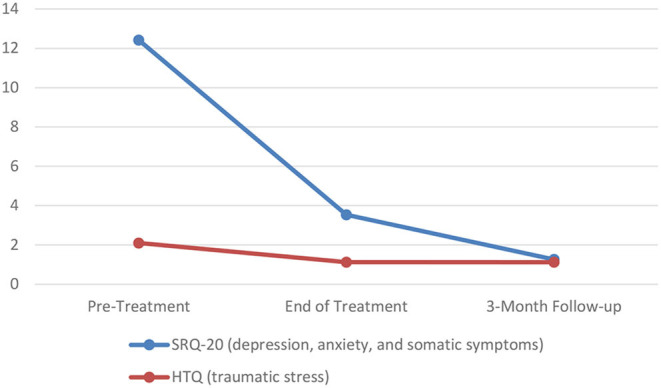
Changes in symptoms over time.

To examine differences in symptoms related to depression and anxiety (SRQ-20) and traumatic stress (HTQ) scores across the three assessment points (baseline, post-treatment, and 3-month follow-up), two separate ANOVAs were conducted (Figure 2). There was a significant effect on SRQ-20 scores over the three time points [*F*_(2, 40)_ = 68.45, *p* < 0.001 partial η^2^ = 0.774]. Pairwise comparisons revealed significant decreases in symptoms related to depression and anxiety from baseline to post-treatment (*p* < 0.001) and post-treatment to 3-month follow-up (*p* = 0.001). Additionally, there was a significant effect on HTQ scores over the three time points [*F*_(1.10, 21.98)_ = 100.51, *p* < 0.001 partial η^2^ = 0.834]. Pairwise comparisons revealed a significant decrease in traumatic stress scores from baseline to post-treatment (*p* < 0.001), but not from post-treatment to 3-month follow-up (*p* = 0.358). It should be noted that the initial decrease in HTQ scores (baseline to post-treatment) was maintained at the 3-month follow-up assessment.

### Treatment Integrity

All five nurses attended every day of the training. On average, nurses completed 60% of homework assignments and earned a 97.5% on quizzes. Nurses completed a fidelity checklist after each group meeting and emailed it to intervention developers to review during supervision. Based on nurse ratings, fidelity to the intervention was very high (Table 4). On average, each group treatment had 3 supervision calls, ranging in length from 15 min to 1 h and 30 min. Supervision calls were shortened or not held due to scheduling conflicts, Internet difficulties, lack of electricity, and difficulty securing an interpreter for the supervision calls.

**Table 4:** Percentage of skills completed by group leaders during group CBT sessions.

**Component skills (number of skills)**	**Group 1%**	**Group 2%**	**Group 3%**	**Group 4%**	**Group 5%**
Introduction (12)	100	100	100	100	100
Psychoeducation (21)	100	100	100	100	100
Behavioral activation (15)	100	100	100	86.67	86.67
Relaxation (21)	100	100	100	100	80.95
Problem solving (18)	100	100	100	100	100
Trauma narrative (20)	100	100	100	100	100
Cognitive restructuring (18)	100	100	100	100	100
Review and planning (14)	100	100	100	100	100
Celebration (5)	100	100	100	100	100

### Group Member Acceptability

Women reported they enjoyed being in the group “very much” (8.89/10) and were interested and engaged in the group content “very much” (9.21/10). Women also reported the intervention was “very helpful” to learn new skills to feel better (9.00/10), and they rated the understandability of group topics as “very understandable” (9.37/10). The majority of group members (96%) believed receiving a colored bead helped them remember the specific skills and found the bracelet helpful. Similarly, most group members (96%) would recommend the COFFEE intervention to future patients and believed fistula nurses should be trained in the program.

### Group Leader Acceptability

At the end of treatment, group leaders rated the acceptability of the group to patients and to themselves. Group leaders rated their responses from 0 (“not at all”) to 10 (“very helpful” or “very much”). Group leaders reported they enjoyed leading groups “very much” (10/10) and women enjoyed being in the group “very much” (9.8/10). Group leaders rated the women as being highly interested and engaged in group topics (9.7/10) and the majority of women understood the group content “very well” (8.8/10). All of the nurses reported giving a colored bead to participants to help them remember the specific skills was helpful. Additionally, all of group leaders would recommend the COFFEE intervention to future patients, would be interested in leading COFFEE groups in the future, and believed fistula nurses should be trained in the program. Lastly, group leaders rated the groups “very effective” (9.1/10).

### Group Member Feedback at 3-Month Follow Up

When asked about the usefulness of the group intervention, 100% of participants thought fistula patients should participate in this program in the future, and 100% agreed nurses should be trained in the intervention. When asked to rate how helpful the intervention was “to learn new skills to feel better,” the women rated the program “very helpful” (9.86/10). When asked about the frequency of using the skills, 81% of women reported remembering the connection among thoughts, feelings, and behaviors, 76% engaged in fun activities, 67% practiced relaxation strategies, 76% engaged in sequential problem solving, and 76% engaged in cognitive restructuring either “3–4 days a week” or “every day.” When asked about the usefulness of the bracelet, 100% thought the bracelet was a helpful reminder of skills, and 67% of women wore the bracelet “3–4 days a week” or “every day.”

## Discussion

We aimed to develop an evidence-based intervention targeting symptoms of depression, anxiety, and traumatic stress in women recovering from fistula repair surgery in the University of Gondar Hospital in Gondar, Ethiopia. We began by conducting a qualitative study of medical staff and women awaiting fistula repair surgery and learned how people in the hospital community understand the mental health needs of women with fistulae. Feedback from medical staff members and fistula patients was used to develop a psychological intervention targeting women recovering from fistula repair surgery (see Table 5). Interviews revealed the interest of both parties—the medical professionals and the patients—in services that will help fistula patients cope with their traumatic stress, anxiety, and depression. In addition, these interviews highlighted several features that would be important for an intervention designed to address mood difficulties in this population. First, feedback from both medical staff and fistula patients suggests that hospital nurses are well-situated to deliver an intervention, given their interest in supporting women's mental health and the comfort that patients feel with them. Second, interviews with medical staff members suggest that the 2 weeks post-surgery, in which women are housed in the hospital while waiting to learn if their surgery was successful, would be an ideal time to deliver an intervention. Third, several interviews stressing the importance of community in the hospital, and also noting the success of group educational sessions already conducted on the unit, suggest that a group format may be preferred. Women are generally housed in the hospital until 8 or 10 patients are ready for surgery, at which point a surgeon comes to the hospital to perform all the surgeries at once; as such, women recover from surgery in groups, and thus would be available for group sessions. Finally, interviews with fistula patients suggested the importance of including a psychological intervention focused on teaching coping strategies, and also recognition of the trauma experienced by many women living with obstetric fistulae. Providing skills and counseling to women on the fistula unit immediately following surgery, through group sessions with nursing staff instruction, may well-enable them to leave the hospital with marked improvements in their physical and mental health.

**Table 5:** Using themes from qualitative interviews to inform intervention structure and components.

**Interview themes**	**Intervention structure**	**Intervention components**
**Staff**		
Stigma of living with fistula	• Group format	• Behavioral activation session • Cognitive restructuring session
Hospital experiences	• Hospital-based • Post-surgery	• Psychoeducation session • Behavioral activation session
Need for mental health intervention	• Hospital-based	• Psychoeducation session
Role of nurses	• Nurses as group leaders	• Celebration session
**Women**		
Coping	• Group format	• Behavioral activation session • Relaxation session • Problem solving session • Trauma narrative
Hospital experiences	• Group format • Nurses as group leaders	• Psychoeducational session
Expectations for their lives following surgery	• Skills bracelet to take home • Discussing use of skills after discharge	• Behavioral activation session • Relaxation • Cognitive restructuring session
Interest in intervention	• Hospital-based	• Behavioral activation session • Relaxation session • Problem solving session • Cognitive restructuring session

We then developed and pilot tested the group-based COFFEE intervention to treat symptoms of anxiety, depression, and traumatic stress in women with obstetric fistulae, based on standard cognitive behavioral approaches to addressing internalizing symptoms in LMIC. COFFEE was developed for nurses to deliver during routine clinical care in the hospital while patients were recovering from fistula repair surgery. The pilot study evaluated the feasibility, acceptability, and clinical benefit of the psychological intervention. We found that nurses were able to implement this program with fidelity; women found intervention groups to be helpful; women's symptoms of depression, anxiety and traumatic stress decreased from baseline (post-surgery) to hospital discharge; and symptom improvement was sustained across a 3-month follow-up interval. Results from this research suggest that women recovering from fistula repair surgery need mental health support, hospital personnel are interested in addressing women's psychological needs, and a group-based intervention holds promise as an approach to decreasing symptoms of mental illness in this vulnerable population.

Data from the qualitative study is consistent with literature suggesting that women with fistulae may benefit from mental health support in addition to medical intervention. For example, in a review of qualitative research with medical personnel and with women with fistulae, Lombard et al. (47) highlighted the need for education and counseling for women post-surgery, in order to support their reintegration into their communities. Likewise, Emasu et al. (24) recommend psychological counseling for women during the recovery period after fistula surgery. A strength of this effort is the inclusion of qualitative data from women with fistulae in addition to data collected from medical personnel on the fistula unit. In fact, Lombard et al. (47) note the importance of gathering the perspectives of marginalized populations rather than relying solely on the institutional perspectives of health care providers in learning about the rehabilitation needs of women after obstetric fistula care. The qualitative data from this study informed the development of a culturally appropriate intervention, consistent with strategies identified by Patel et al. (48) for addressing the barriers to psychological care in low-income countries.

This pilot study of the COFFEE intervention suggests that a structured, cognitive behavioral intervention may be appropriate for addressing symptoms of depression, anxiety, and traumatic stress in women recovering from fistula repair surgery. Because we learned through interviews about the importance of the social support offered by other patients on the fistula unit, we elected to use a group approach in developing the COFFEE program, which is, to our knowledge, the first example of a cognitive behavioral intervention for this population that is delivered in groups. Ojengbede et al. (28) have highlighted the value of a group format in their study of women who shared their experiences with fistula while waiting for fistula repair surgery; while there was no control group in this study, the authors conclude that the social support and sharing encouraged through the group format helped improve women's psychological wellbeing. The focus on social support offered in a group format is consistent with literature suggesting the importance of social roles during women's rehabilitation process following fistula repair surgery (47, 49), and also capitalizes on the communal culture of rural Ethiopia, from where many of the patients on the unit traveled from for care in the city of Gondar (50). In addition to providing fellowship, the group format is inherently less expensive than individual interventions (28), which are often less feasible given the dearth of mental health practitioners in LMIC (51).

Given the positive results of the current trial, a randomized controlled trial comparing patients who received COFFEE to those who did not after fistula repair surgery will help determine if the COFFEE intervention helped decrease symptoms of anxiety, depression, and traumatic stress above and beyond the benefits of the fistula repair surgery itself. A CBT protocol designed to address common mental health problems for women with obstetric fistulae and intended for efficient training and ease of implementation by nurses during routine post-operative care could help extend evidence-based treatments to women in Ethiopia and other LMICs who would otherwise not receive psychological care.

### Limitations

There are several notable limitations to this work. First, for the qualitative study, practical constraints were such that we were only able to conduct ten interviews with hospital staff and six interviews with fistulae patients. As such, we are not confident that we reached thematic saturation. Nevertheless, consistent with O'Reilly and Parker (52), we maintain that our inability to claim thematic saturation does not diminish the value of our data but highlights the need for further research in this area.

Second, because we present data from an open trial pilot study, we are not able to determine whether the decrease observed in women's symptoms of depression, anxiety and traumatic stress are due to their participation in the COFFEE intervention, or whether they would have experienced improvements in mental health because they had received surgery to correct a debilitating and stigmatizing medical condition. In fact, studies of women with fistulae worldwide suggest that mental health improvements accompany surgical repair even when no mental health support is delivered. Specifically, in a small study (*N* = 28) of women in Tanzania, Wilson and colleagues (17) reported significant improvements in symptoms of depression from pre-surgery to 3-months post-surgery, especially among women with successful surgical outcomes (i.e., continent of urine and/or feces). Likewise, Belayihun and Mavhandu-Mudzusi (23) followed a larger group (*N* = 200) of women in Ethiopia from pre-surgery to post-surgery and found rates of elevated depression scores dropped from 91 to 27% at 6-month follow-up, while rates of elevated anxiety scores dropped from 79 to 26%. Finally, in Uganda, Ayadi et al. (22) followed women (*N* = 60) from pre-surgery across a 12-month follow-up interval and found that symptoms of depression and traumatic stress decreased substantially over time, although no psychological interventions were delivered. Although in all of these studies outcomes were better for women whose fistulae were successfully repaired, some studies find that women experience improvements in social support and quality of life even when surgical outcomes are disappointing (18, 47). A future randomized controlled trial will enable us to determine whether the improvements we noted in women's symptoms of depression, anxiety and traumatic stress are due to their participation in the COFFEE intervention, or to the surgical repair itself.

Third, the supervisory model used in this study was limited, and, as a result, it is difficult to determine whether or not the COFFEE intervention was delivered with fidelity. While supervision was provided to group leaders via Skype, several barriers (i.e., poor internet quality, language barriers, differences in time zones) made it challenging to conduct supervisory sessions regularly, and some group leaders received more support than others. Moreover, fidelity ratings from supervisors are not available, and it is possible that group leaders' fidelity ratings may in part reflect their desire to please the research team. Murray et al. (53) describe their use of an apprenticeship model in implementing mental health interventions in LMIC where task shifting (i.e., training non-mental health professionals to deliver mental health interventions) is essential to meet the needs of the local population. This model uses trainers, who are often not local but are experts in the target intervention, local supervisors, and then local counselors who deliver the interventions to the target population. For example, in an effort to deliver a group interpersonal psychotherapy intervention targeting depressive symptoms in adolescents in Uganda, Bolton et al. (54) trained staff in Uganda to serve as local supervisors to group leaders; experts in the intervention from the United States provided weekly supervision to the local supervisors, who provided direct support to the group leaders. In fact, adequate supervision has been identified as an essential component in supporting intervention fidelity, and in sustaining mental health interventions over time (53). The use of such an apprenticeship model for the COFFEE intervention would have mitigated the supervisory challenges encountered in this trial, and would have supported long-term use of this intervention in the fistula hospital.

Finally, we did not collect demographic, social, and medical data that would have helped us to interpret our findings. In a meta-analysis of the prevalence of depression among women with obstetric fistulae in Africa, Duko, Wolka, Seyoum and Tantu (21) noted a number of factors that affect mental health outcomes in this population that unfortunately were not assessed in our study. For example, we did not collect data on the number of children that study participants had, although rates of depression in women with fistulae may be affected by whether or not they have a living child (18). Additionally, we did not collect data on women's social support or quality of life, although research indicates that the degree of social support women experience both pre- and post-surgical repair may impact their depression scores (12), and that many women post-surgical repair experience significant improvements in quality of life (47). Moreover, we did not systematically collect data from study participants regarding the outcome of their fistula surgery, although we know anecdotally that the surgery for two of the women in our pilot study was not successful. Although 84–94% of fistula repair surgeries are successful (1), the literature suggests that many women who remain incontinent of urine or feces following surgery experience residual symptoms of depression (17, 18).

### Future Directions

In order to determine if the COFFEE intervention decreased symptoms of anxiety, depression, and traumatic stress, a randomized clinical trial is necessary to resolve if the decreases in symptoms in the current study were attributable to the fistula repair surgery or to the COFFEE intervention. A full, randomized clinical trial, using a larger sample and a longer follow-up interval, would control for the effects of the fistula repair surgery as well as for the passage of time. We would recommend using the apprenticeship model (53) to train both lay counselors and lay supervisors, in order to address some of the difficulties encountered around supervision of group leaders during the pilot study. If the COFFEE intervention adds clinical benefit above the fistula repair surgery alone, attention should be placed on sustaining the intervention without grant funding.

## Data Availability Statement

The raw data supporting the conclusions of this article will be made available by the authors, without undue reservation.

## Ethics Statement

The studies involving human participants were reviewed and approved by the Wellesley College Institutional Review Board and the University of Gondar Institutional Review Board. The patients/participants provided their written informed consent to participate in this study.

## Author Contributions

TG and AU: conceptualization and supervision. TG, AU, MM, TM, and GA: data collection. TG, AU, TM, MP, and KB: data analysis. TG, AU, and CZ: writing—original draft preparation. TG, AU, MM, TM, GA, MP, CZ, and KB: writing—review and editing. TG: funding acquisition. All authors have read and agreed to the published version of the manuscript.

## Funding

This research was funded by the Empowering Children for Life: The Robert S. and Grace W. Stone Primary Prevention Initiatives at the Wellesley Centers for Women, Wellesley College, Wellesley, MA.

## Conflict of Interest

The authors declare that the research was conducted in the absence of any commercial or financial relationships that could be construed as a potential conflict of interest.

## Publisher's Note

All claims expressed in this article are solely those of the authors and do not necessarily represent those of their affiliated organizations, or those of the publisher, the editors and the reviewers. Any product that may be evaluated in this article, or claim that may be made by its manufacturer, is not guaranteed or endorsed by the publisher.
